# Current and Future Perspectives for Improving Ovarian Tissue Cryopreservation and Transplantation Outcomes for Cancer Patients

**DOI:** 10.1007/s43032-021-00517-2

**Published:** 2021-03-31

**Authors:** Sanghoon Lee, Sinan Ozkavukcu, Seung-Yup Ku

**Affiliations:** 1 Moores Cancer Center, University of California San Diego, San Diego, CA USA; 2 Department of Obstetrics and Gynecology, Korea University College of Medicine, 73, Inchon-ro, Seongbuk-gu, Seoul, 02841 Republic of Korea; 3 Center for Assisted Reproduction, Department of Obstetrics and Gynecology, Ankara University Faculty of Medicine, Ankara, Turkey; 4 Department of Obstetrics and Gynecology, Seoul National University College of Medicine, Seoul, Republic of Korea

**Keywords:** Fertility preservation, Ovarian freezing, Autologous transplantation, Cancer treatment, Oncofertility, Primary ovarian insufficiency

## Abstract

Although advances in cancer treatment and early diagnosis have significantly improved cancer survival rates, cancer therapies can cause serious side effects, including ovarian failure and infertility, in women of reproductive age. Infertility following cancer treatment can have significant adverse effects on the quality of life. However, established methods for fertility preservation, including embryo or oocyte cryopreservation, are not always suitable for female cancer patients because of complicated individual conditions and treatment methods. Ovarian tissue cryopreservation and transplantation is a promising option for fertility preservation in pre-pubertal girls and adult patients with cancer who require immediate treatment, or who are not eligible to undergo ovarian stimulation. This review introduces various methods and strategies to improve ovarian tissue cryopreservation and transplantation outcomes, to help patients and clinicians choose the best option when considering the potential complexity of a patient’s situation. Effective multidisciplinary oncofertility strategies, involving the inclusion of a highly skilled and experienced oncofertility team that considers cryopreservation methods, thawing processes and devices, surgical procedures for transplantation, and advances in technologies, are necessary to provide high-quality care to a cancer patient.

## Introduction

A large number of young women of reproductive age are diagnosed with cancer each year. In 2018, approximately 8.6 million women worldwide struggled with a malignancy [1, 2]; although most of these women were of advanced age, 10% were under the age of 45 [3]. In 2020, an estimated 89,500 cancer cases and 9270 cancer deaths occurred among adolescents and young adults aged 15 to 39 years in the USA [4]. Early diagnosis and follow-up methodologies, especially chemotherapy and radiotherapy, have increased the survival rates of these patients [1]. However, both of these treatments can cause loss of ovarian function and primary ovarian insufficiency (POI) due to follicular depletion [5, 6], with alkylating agents, such as cyclophosphamide, known to be severely gonadotoxic [7]. In addition, ovarian surgeries to treat endometriosis or other benign ovarian tumors, and the cytotoxic agents used to treat some benign diseases, have also been associated with fertility loss [8, 9].

In recent years, there has been a sharp increase in the number of women diagnosed with cancer seeking to preserve their future fertility [10]. Embryo and oocyte cryopreservation are well-established methods of fertility preservation. Early referral to reproductive specialists is crucial for patients of pre-pubertal and childbearing age with cancer [11–13]. Ovarian tissue cryopreservation and transplantation (OTC-T) is an essential option for fertility preservation, particularly for post-pubertal women with cancer who require immediate chemotherapy or who are not eligible for ovarian stimulation; however, for pre-pubertal female patients, it is the gold standard in fertility preservation [14].

A recent study evaluating 364 women who had undergone ovarian tissue cryopreservation (OTC) showed that more than 95% had experienced long-term restoration of ovarian function [15]. Since 2004, when the first human live-birth relating to ovarian cortex auto-transplantation was recorded, technique improvements have resulted in an estimated live birth rate between 35 and 40% [16]. Although the exact number of OTC, or OTC-T cases is unknown, and given that the effectiveness of the procedure cannot be calculated definitively, it is estimated that, worldwide, more than 130 children, with a 30% conception rate, have been conceived following ovarian tissue transplantation (OTT) [10, 15, 17]. OTC and re-transplantation were previously considered “experimental”; however, following heavy debate, and given the increasing number of reported live births and experience in surgical and laboratory techniques, it is now registered by major societies of reproduction as an acceptable procedure for fertility preservation [18, 19]. A key drawback of this procedure is the risk of malignant contamination on the ovarian graft following auto-transplantation. To date, not a single case of relapse due to malignant cell debris in transplanted tissue has been reported in humans; however, some studies have indicated an increased risk of hematological malignancy relapse in xenotransplantation models involving only immunosuppressed animals [20, 21].

Recovery of endocrine function and fertility through OTC-T may improve the quality of life of women of reproductive age following cancer survival [14]. This review discusses novel methods and strategies to improve the outcomes of OTC-T; thus, allowing physicians to choose the most appropriate fertility preservation technique for their patients.

### Ovarian Toxicity Caused by Cancer Treatment

Although advances in chemotherapy and radiation therapy have greatly improved the effects of cancer treatment and the survival rate of cancer patients, gonadal damage remains a serious complication. The incidence of chemotherapy-induced amenorrhea reportedly ranges from 53 to 89% in patients with breast cancer [22]. Iatrogenic POI caused by conventional chemotherapy treatments depends on the type, dosage, and duration of chemotherapy drugs, as well as the age of the patient [23]. Primordial follicles decline with increasing age, with a higher risk of gonadal damage and infertility in older patients. Since most cancer patients are treated with multiple chemotherapy drugs, it is difficult to assess the extent of reproductive damage caused by each specific agent [24, 25].

Anticancer drugs primarily exert their gonadotoxic effects through the direct induction of DNA double-stranded breaks, resulting in the activation of apoptosis and/or autophagy-related pathways [26–29]. Second, anticancer drugs can indirectly cause primordial follicle loss via microvascular and stromal damage through ischemia, necrosis, or inflammation [26, 28, 30]. Third, studies have shown that anticancer drugs can activate the PI3K/AKT/FOXO3a pathway, leading to follicular loss via the activation of primordial follicles [29, 31, 32]. Follicle activation and ovarian reserve burn-out are also potentially important mechanisms of follicle loss post-transplantation of OT grafts [33].

Primordial follicles are very sensitive to radiation, and the degree of damage to the ovaries depends on the total dose, field of treatment, fractionation schedule, and age at the time of treatment [34]. Radiotherapy-induced amenorrhea can occur when pelvic or whole abdominal areas are exposed to a radiation dose ≥ 6 Gy in adult women, ≥ 10 Gy in post-pubertal girls, and ≥ 15 Gy in prepubertal girls [34–36].

### Comparison of OTC Techniques: Slow Freezing vs Vitrification

The age of the patient, presence of a partner, treatment method, and possibility of treatment delay should all be considered when considering fertility preservation. Cryopreservation of both embryos and oocytes requires a period of approximately 10–14 days from the onset of menstruation for ovarian stimulation; however, since all oocytes and embryos are frozen at the end of a cycle, random start protocols may be used for controlled ovarian stimulation. Although embryo cryopreservation was considered to be the best-established fertility preservation technique worldwide, in jurisdictions where each partner has equal legal rights over an embryo, their utilization is subject to each parent’s consent. Furthermore, divorce rates are known to increase in families where one partner is receiving cancer treatment, especially in women [37]. As such, embryo cryopreservation can become an option that limits the freedom of a divorced woman seeking to reconsider her fertility preferences in the future, as accessing the cryopreserved embryos is dependent on her ex-partner’s consent. Since oocyte cryopreservation is considered a standard procedure in modern assisted reproductive technologies, it should be offered as the first option for post-pubertal women applying for fertility preservation [34, 36].

As OTC has the advantage of not requiring a sperm donor or ovarian stimulation, it is the only possible choice for pre-pubertal girls and patients who cannot postpone cancer treatment for ovarian stimulation. In contrast to freezing individual oocytes or embryos, OTC can effectively preserve hundreds of primordial follicles simultaneously [14]. This technique can be performed using slow freezing or vitrification. Using the slow freezing method, OT is frozen slowly in a controlled manner down to −140 °C, and then stored at −196 °C, in liquid nitrogen. Using this technique, no serious tissue deformation is observed; however, there is a risk of ice crystal formation causing mechanical damage to the cells [38]. In contrast, vitrification involves instantaneous solidification of the solution, with viscosity maintained using a high concentration of cryoprotectant agents [16]. Notably, this technique has a low risk of ice crystal formation, reduced handling time, and inexpensive equipment.

A recent systematic review and meta-analysis of 14 experimental studies compared vitrification with slow freezing for OTC [39]. In pooled analyses, no significant difference in follicular density or proportion of intact primordial follicles was observed between the two methods; however, vitrification was associated with significantly less damage to follicular DNA and better preservation of stromal cells. This review also emphasized the diversity of the vitrification protocols used in the studies, thereby highlighting the lack of standardization [39]. Some studies suggested that regardless of the freezing method, type of follicle, or species involved in an experiment, vitrification resulted in greater damage to the follicles than slow freezing [40]. In addition, the ability of frozen thawed cortical tissue to produce anti-Müllerian hormone (AMH) in tissue cultures was superior after slow freezing [41]. According to previous studies, only a small percentage of children have been conceived following transplantation of vitrified-warmed OT [15, 42–44]. Furthermore, for vitrification, the volume of the sample must be reduced in order to reach the maximum rate of heat exchange. Considering the size of OT biopsies, obtaining very small fragments may hinder long-term cryo-storage methods and jeopardize surgical procedures during re-transplantation. Even minimally fragmented ovarian pieces are thousands of times larger than the volume of an oocyte. Practically, smaller fragments increase the number of pieces to be vitrified, and an operator must conduct each step, namely, equilibration, loading, and vitrification, individually for each fragment, each of which requires an optimum, yet limited, duration in the highly concentrated and toxic equilibrium and vitrification media [45]. This method significantly extends the total duration of the freezing process, thereby increasing the risk of toxicity due to a high CPA concentration. Slow freezing is currently considered a more appropriate method for OTC than vitrification [46]. Considering the limited comparisons between the effects of vitrification and slow freezing on OT, further studies investigating the efficiency of vitrification are required.

### Potential Considerations for Improving the Outcomes of OTC

Considering that majority of pregnant women reported in the literature have been under the age of 30 at the time of cryopreservation, patient age is a key factor for predicting its success. Thirty-five years of age is generally considered the upper limit for OTC, as primordial follicles are primarily preserved during this procedure, and their number decreases significantly with age [47]. AMH levels and antral follicle count can be used to test the ovarian reserve; patients should be informed about their chances of future pregnancy prior to fertility preservation [48]. Recently, many suggestions regarding the criteria for selecting patients for ovarian cryopreservation have been proposed [49]; however, some criteria require ethical consideration.

#### Cryoprotectants

Multiple cryoprotectants are used for OTC, with several of those used for slow freezing human OT been previously studied, including glycerol, ethylene glycol, dimethyl sulfoxide (DMSO), and propanediol. Follicle survival was evaluated after thawing fragments of frozen ovarian cortical tissue with cryoprotectants. The highest and lowest follicle survival rates were obtained with ethylene glycol and glycerol, respectively [50]. Although the most commonly used cryoprotectants are ethylene glycol and DMSO, propylene glycol is also used by some centers [51–53]. One vitrification protocol used 20% DMSO and 20% ethylene glycol, while another used 10% DMSO and 26% ethylene glycol, resulting in a higher proportion of primordial follicles [54]. DMSO, a low molecular weight organic molecule, rapidly penetrates cell membranes and further reduces intracellular ice nucleation, which is generally combined with non-penetrable sucrose [55]. Although used frequently in vitrification protocols, DMSO is usually combined with ethylene glycol and propanediol due to toxicity concerns [56, 57]. DMSO has recently been extracted from some oocyte/embryo vitrification solutions, in which case, a combination of ethylene glycol and propanediol is used [58–60]. Although sucrose is the most commonly used non-penetrating agent in freezing and thawing processes, trehalose has been used as its alternative in recent years [61]. Cryopreservation followed by transplantation is affected by the clinical environment; most studies have evaluated the efficiency of OTC after warming or in vitro culture [54]. Further research is needed to determine the most efficient protocol for vessel formation and cell proliferation after OTC-T.

#### Anti-apoptotic Agents

Other key factors that ensure the survival of OTC-T are revascularization and apoptosis prevention. Several studies have reported that increased apoptotic follicles were observed shortly after OTT [62–64]. Sphingosine-1-phosphate (S1P), an anti-apoptotic substance in oocytes [65, 66], and ceramide play central roles in apoptosis. S1P inhibits ceramide, which induces cell cycle arrest and promotes apoptosis [67]. S1P has been shown to protect vitrified ovarian grafts from ischemic reperfusion injury and promote neo-angiogenesis in ovarian transplants [68, 69]. However, we found that S1P did not help preserve, or increase the proliferation of, follicles, nor did it protect against DNA damage during the freezing-thawing process (unpublished results). In contrast, Z-VAD-FMK administration improved follicle preservation and follicular cell proliferation, also preventing DNA damage during the freezing-thawing process [70]. Although these substances are known to contribute to follicle protection by reducing apoptosis in transplanted OT, their routine use is not possible in contemporary practice; further investigation is warranted.

#### AMH

AMH, another promising agent for fertility preservation, belongs to the transforming growth factor (TGF)-beta family of proteins, and plays a key role in controlling sexual differentiation and follicular genesis. Although serum AMH has long been used in reproductive biology as a key marker of ovarian reserve, it has also recently been investigated as a protective agent [71, 72]**.** Administration of recombinant AMH inhibits the initiation of primordial follicle recruitment [73, 74]; recently, co-administration of AMH and chemotherapy agents has been shown to protect the ovarian reserve by suppressing primordial follicle recruitment [75].

One challenge in OTC is mass primordial follicle loss in the OT immediately after transplantation, resulting in the shorter longevity of transplanted ovarian function [64]. Using AMH to inhibit primordial follicle recruitment has proven useful in reducing this initial follicular loss; co-transplantation of the graft with exogenous endothelial cells engineered to produce AMH in situ significantly decreased primordial follicle loss in human xenotransplants [76, 77]. These studies suggest that it may be possible to protect the ovarian reserve from gonadotoxic drugs; further human clinical trials are warranted [78].

#### Slush Nitrogen (SN)

Liquid nitrogen is limited in its efficiency as a coolant due to the Leidenfrost effect, namely, it boils immediately upon contact with a warmer object, forming insulating nitrogen gas. Recently, SN has been proposed as a new agent for increasing the cooling rate, avoiding the Leidenfrost effect, and facilitating the use of low concentrations, or reduced exposure times, of cryopreservation agents (CPAs) [79]. Reportedly, fresh and SN-vitrified mouse oocytes develop to the same blastocyst stage and produce similar proportions of healthy offspring. SN has improved the vitrification outcomes of both human oocytes and OT, including recovering healthy oocytes, granulosa cells, and stromal cells via an increased cooling rate [80–83]. At the tissue level, where larger volumes of samples are prepared, faster cooling rates are required to maintain vitrification; SN can be used as an alternative method through which to rapidly reduce the temperature.

#### Laser-assisted Thawing

Successful vitrification depends largely on the rate of warming, rather than the type and concentration of cryoprotective agent, and successful osmotic dehydration before cooling, to avoid the re-crystallization of water in the thawing cycle, when very rapid warming is essential. A recent study reported high oocyte and embryo survival rates following vitrification, without cryoprotectant permeation after thawing via ultra-fast warming using an infrared laser pulse [84]. Another study used a laser beam to dehydrate the blastocoel before vitrification, finding significant improvements in clinical outcomes due to decreased ice recrystallization [85].

#### Cryopreservation as a Method to Reduce the Risk of Cancer Cell Reimplantation

OTC may be possible for patients with aggressive cancer who require immediate chemotherapy; however, contaminating OT with malignant cells is a major concern when considering this technique [86, 87]. A small number of malignant cells may be present in OT, with relapses following transplantation having been observed in both human and mouse hematologic malignant tumor models [88, 89]. Although no case in which a relapse occurred following OT auto-transplantation has been reported, animal studies have shown that hematological malignancies can settle in the ovary, potentially causing relapses or metastases under appropriate conditions. These results were predominantly derived from xenografting studies using immunosuppressed mice. Malignant cells in grafted OT may never undergo activation or may be eliminated due to the patient’s newly formed immune system following bone marrow transplantation. Therefore, although the experimental xenograft model highlights the importance of remaining alert, it may be inadequate for demonstrating the accurate clinical situation. Therefore, despite two recent publications reporting successful fertility preservation after ovarian transplantation in acute leukemia, this technique may not be suitable for women with ovarian or hematologic malignancies [90, 91]. In the future, reproductive function or fertility may be preserved in these patients via the in vitro maturation (IVM) of oocytes and artificial ovary techniques [87]. Some studies have proposed that even if ovarian function is impaired, OT can be cryopreserved after undergoing an initial chemotherapy process to reduce the risk of cancer contamination [21, 92]. Reportedly, in vitro incubation with YAP/TAZ inhibitor verteporfin prior to auto-transplantation eliminates rhabdomyosarcoma and leukemia cells that have metastasized to the OT [93]. These studies strongly recommend the wide screening of tissue samples containing fragments intended for transplantation using both histological examinations and possible molecular biomarkers. Polymerase chain reaction, flow cytometry, or immune labeling should be used to rule out tumor contamination in transplanted tissues, especially in malignancies prone to ovarian metastasis [94, 95].

### Surgical Considerations to Improve the Outcomes of OTT

#### Introduction to Surgical Techniques

Prior to receiving cancer treatment, OTC-T in women diagnosed with cancer is an effective option for preserving fertility and restoring reproductive endocrine function [96]. Various surgical techniques have been introduced to transplant human OT following cryopreservation, including open laparotomy, mini-laparotomy, laparoscopy, and robot-assisted transplantation [97]. The first ovarian transplantation with cryopreserved OT was performed in 1999 [98, 99]. In 2004, the first successful live birth was reported following the transplantation of OT that was cryopreserved using the slow freezing method [100]. In recent years, the number of pregnancies and births following cryopreserved OTT using different surgical techniques has steadily increased [17]. Robot-assisted transplantation may have several advantages over laparoscopic transplantation, including precision, more delicate graft handling, and reduced time from tissue thawing to transplantation [101]. Physicians should select the most suitable technique through which to maximize OTT outcomes by considering their own clinical experience and animal experiments.

As the decision to perform ovarian cryopreservation is often made clinically when patients have limited time before starting chemotherapy, a laparoscopic approach, involving minimally invasive surgery, is a very useful technique. One of the most important advantages of laparoscopic surgery is that patients recover very quickly, enabling them to promptly start chemotherapy [97]. In particular, a single-port laparoscopy is widely used in surgical gynecology and is associated with a low rate of complications following OT extraction [102].

#### OT Extraction and Surgical Tools

Various pinions exist on the volume of tissue that should be extracted when collecting OT. Large samples have the advantage of allowing for repeat transplantations, which potentially maintains reproductive and endocrine ovarian function longer. Different strategies may be considered depending on a patient’s situation. If aggressive chemotherapy with alkylating agents, pelvic irradiation, or high-dose chemotherapy is necessary prior to bone marrow transplantation, large amounts of OT should be removed before ovarian failure. However, keeping more OT in situ may be a more desirable strategy when less toxic chemotherapy is scheduled. For OT excision, ovarian cortical biopsy, or partial or complete oophorectomy, may be performed [103]. According to the von Wolff group, 50% resection of the ovary may be sufficient for cryopreservation [104]. Following cancer treatment completion, frozen OT can be prepared and grafted onto the surface of the remaining ovary or the pelvic peritoneum [105]. Although frozen-thawed OT is orthotopically transplanted in most cases, heterotopic transplantation into the subcutaneous space of the abdominal wall or the forearm can be considered in unavoidable circumstances [106, 107].

Generally, scissors or a scalpel without coagulation are recommended to use when extracting and transplanting OT, as electrocoagulation may damage OT and reduce ovarian reserve [108, 109]. Using sutures or fibrin adhesives on the ovarian surface may be considered during tissue transplantation after weighing the potential risk of secondary bleeding. In pre-pubertal patients whose treatment regime contains highly toxic interventions, such as alkylating agents or radiotherapy, unilateral ovarian extraction should be considered following parental consultation. As the organ size is significantly smaller in this age group, optimal fertility preservation can be achieved using one whole ovary.

#### Potential Sites for Transplantation

Potential sites for transplanting frozen-thawed OT are (i) on the remaining menopausal ovary [110], (ii) into the ovarian ligament [111], (iii) a pre-prepared peritoneal pocket [91], or (iv) heterotopic transplantation [107]. Potential advantages of an ovary as a transplantation site include that the tissue was originally collected from the same site; hence, transplanted samples could easily be located and revived. However, the risk of bleeding and the possibility of ovarian trauma due to sutures must be considered; the peritoneal pocket may be another option if the remaining OT is not sufficient to receive the transplant. Although heterotopic transplantation has the advantage of not requiring abdominal surgery, all births reported thus far have been from orthotopic transplants; spontaneous pregnancies are difficult to predict, and in vitro fertilization is required [14, 46]. Reports of spontaneous pregnancies and live births following heterotopic transplantations have caused controversy regarding stem cell migration between tissues. Further basic and clinical studies are required to elucidate these mechanisms [112]. Heterotopic transplantation is considered a rarely applied method, as multiple physical and biological requirements related to OT are not met via this approach. In animal studies, offspring are reportedly obtained from heterotopic transplants placed close to the cutaneous area [113, 114]. Additionally, oocytes and embryos have been obtained in humans as a result of subcutaneous transplantation [115]. Although it may be controversial to classify transplants placed intraabdominally into the peritoneal wall as heterotopic, pregnancy and live births have been obtained using assisted reproduction in this region [116]. Heterotopic transplantation may be suggested if the pelvic region is deemed unsuitable for transplantation due to scars following radiotherapy.

### Current and Future Perspectives on Fertility Preservation

#### Whole Ovarian Transplantation

Whole-ovarian transplantation enables immediate revascularization with blood vessel anastomosis, significantly reducing the risk of ischemic injury [117]. Furthermore, whole cryopreserved ovary transplantation may reduce the risk of ischemic damage; however, cryopreserving a large quantity of intact ovary is challenging due to difficulties in dispersing a sufficient amount of cryoprotective agent throughout the large tissue mass, and the potential injury caused by ice formation in the blood vessels [118]. Considering that human ovarian arteries and veins have a diameter of approximately 0.5 and 3 mm, respectively, vascular anastomosis, a key technical issue, must be considered [119]. Whole ovary cryopreservation and transplantation have been successfully achieved in several experimental animal studies [120, 121]. One group concluded that vitrification appeared to be more effective than conventional freezing for whole-ovary cryopreservation. However, another study reported that conventional slow freezing of ovarian cortical strips was more suitable than any other method of whole ovary cryopreservation [122]. Although obstacles and technical difficulties remain [123], human whole ovary cryopreservation and transplantation are encouraged, with future studies likely to solve a majority of current issues [124].

#### IVM

IVM has been extensively applied to oocytes obtained from women with polycystic ovarian syndrome [125]. This method involves immature oocyte retrieval from ovaries and either cryopreservation at an immature stage or at a post-IVM matured stage [126]. Both OTC and IVM can be applied to patients with cancer without delaying cancer treatment, including for prepubertal girls and those who need immediate chemotherapy. Moreover, in cancer patients who lack sufficient time for an IVF cycle prior to chemotherapy or radiation therapy, immature oocyte collection may be a promising alternative. Although many researchers aim to achieve improved outcomes by combining IVM of oocytes and vitrification, no live births have been reported from an IVM program in patients with cancer [125, 127]. A key concern, namely, the auto-transplantation of malignant cells in women who have recovered from cancer, may be eliminated by isolating ovarian follicles from OT and maturing them in vitro [122]. Although oocyte cryopreservation with IVM is still considered an experimental technique [125] that requires further technical improvements, it could potentially be used for fertility preservation in the near future.

#### Artificial Ovaries

Studies examining the use of artificial ovaries, or scaffolds, in ovarian transplantation are increasing. The aim of transplanting OT with a scaffold may be to accelerate tissue vascularization through the release of various bioactive substances [128, 129] or to optimize tissue transplantation by attaching it to the scaffold using laparoscopic or robotic surgery [130]. Today, it is most commonly used to enable the transplantation of various substances or accessory cells, such as stem cells, together with OT [131–135]. In addition, the technique may be used to develop follicles under in vitro conditions, thereby avoiding tumor contamination or placing these isolated follicles in the transplantation area with the assistance of scaffolds. An artificial ovary can be used to mature oocytes through a multistep process, including sequential in vitro culturing of oocytes, isolated follicles, and OT [136–145]. Multiple studies have shown that the live birth rate using this option is comparable to that of conventional IVF [146, 147]. Moreover, ovarian follicles cultured in vitro can be reimplanted within a 3D bio-degradable microenvironment. To date, animal studies have demonstrated that this approach restores endocrine function, also enabling in vivo follicular development and successful pregnancy; however, no successful human trials have been reported [138–140, 142]. Developing techniques through which to increase follicular recovery rate and optimize scaffold design, as well as transplantation techniques to prevent postoperative ischemia, and genetic safety considerations, are all necessary for safer and more consistent human clinical applications [136].

#### Stem Cells

Recent stem cell studies have investigated the use of ovarian stem cells in fertility preservation. Tilly et al. reported the successful detection and isolation of ovarian stem cells in animals and humans [137, 141]. Furthermore, studies investigating egg-producing stem cells isolated from women’s ovaries have observed that these cells differentiate into young oocytes [137]. Oocytes differentiated from ovarian stem cells (OSCs) retrieved from mice were suitable for fertilization and implantation, as evidenced by embryo development and live births [143]. This discovery suggests that therapeutic manipulation of adult stem cells can potentially overcome infertility and prevent ovarian failure. Stem cells may be an option for pre-pubertal girls and women with diverse cancer-associated infertility conditions. However, due to the insufficient clinical application of OCSs in human-assisted reproduction, difficulty in detecting OCSs due to their scarcity, and ethical issues associated with using oocytes and embryos, this technique is not commonly used in clinical practice, especially in cancer patients [148–150].

A recent study investigated the effects of adipose-derived stem cells (ASCs) on OTT using an animal model [151]. High ASC concentrations have been shown to increase the human vessel area over time. The ability of ASCs to stimulate human angiogenesis through differentiation and growth factor secretion appears to depend on both cell concentration and time. ASCs grown using a fibrin scaffold served as a substrate to prepare the grafting site over 14 days, also enhancing vascularization following transplantation of human OT. Promoting revascularization by combining OT with angiogenic factors or pro-endothelial stem cells is another approach [152–154]. Further studies are required to implement these approaches in human practice.

#### Substances for Revascularization

A key factor affecting the duration of ovarian graft function is the number of surviving oocytes following freeze-thawing and revascularization. Ischemia is a major cause of follicular loss following transplantation, with reoxygenation taking approximately 4–5 days [155, 156]. Tissues predominantly depend on anaerobic metabolism early in the post-transplantation period; the shift to aerobic metabolism occurs when oxygenation is provided by neo-vascularization. This is evidenced by the microdialysis experiments performed by Cacciottola et al., in which ischemia was not the only factor in damage, with oxygenation following neo-vascularization potentially triggering the formation of reactive oxygen radicals and contributing to tissue injury during the late stage of graft take [157]. Following the transplantation of frozen/thawed OT into SCID mice, approximately 28% of primordial follicles survived the procedure; the remaining follicles died due to ischemic damage [62]. Another study demonstrated that the transplantation site could be treated with vascular endothelial growth factor (VEGF) [158] and stromal cells enriched in CD34 cells to improve angiogenesis [159]. Combined VEGF and bFGF administration induced angiogenesis, reduced apoptosis and fibrosis, and increased the survival of transplanted human OT in a rabbit model [160]. VEGF coupled with FGF2 promoted revascularization and significantly increased the survival rate of transplanted cryopreserved OT, compared with untreated controls, in a mouse model [161]. Treatment with melatonin, or OT incubation with hyaluronan-rich biological glue, in addition to VEGF-A and vitamin E may improve graft survival [162]. In another study, Zhang et al. supplemented the freezing medium with FSH, resulting in increased revascularization and survival of ovarian grafts following vitrification in mice [163].

## Conclusions

**Fig. 1:**
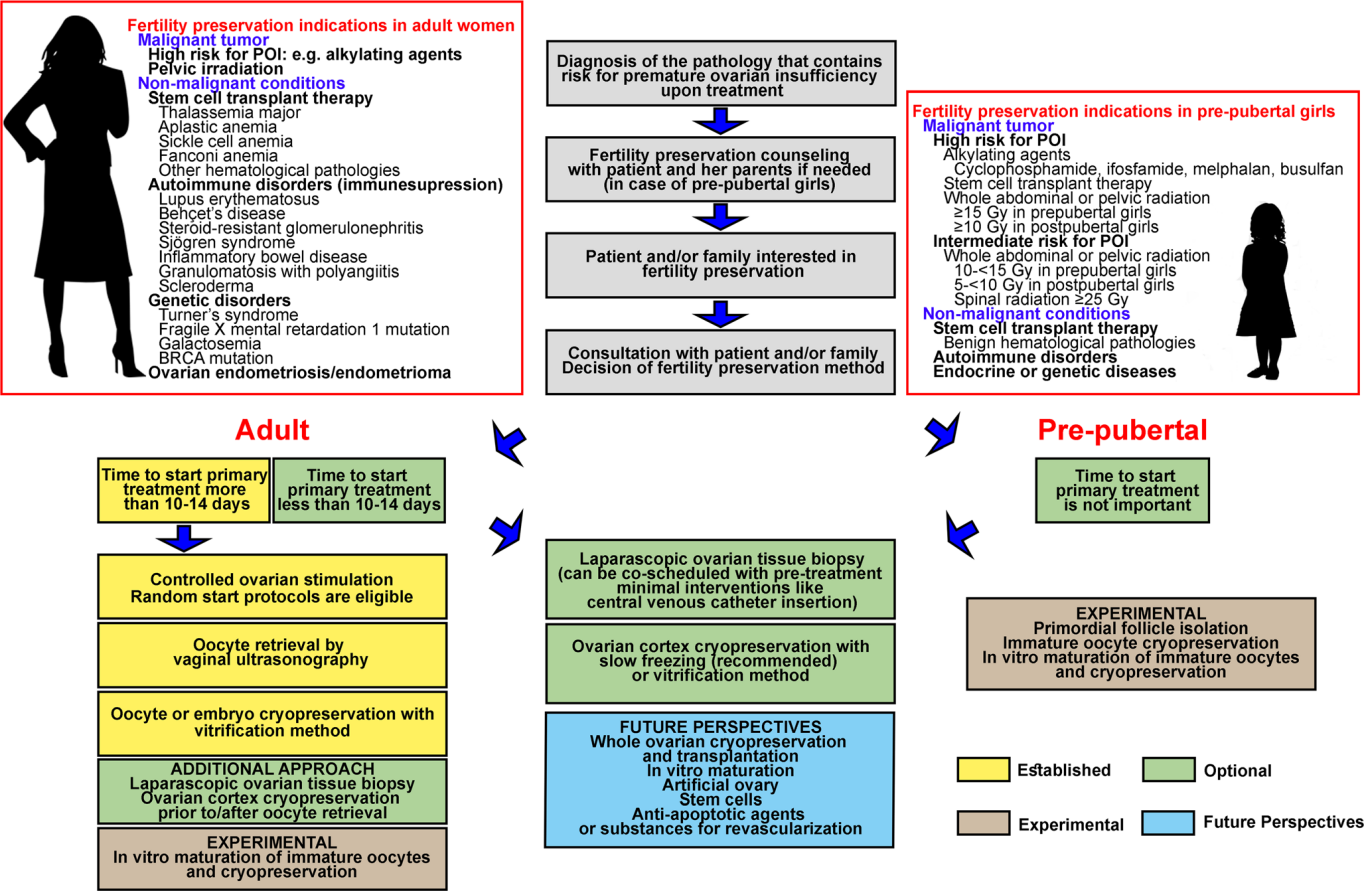
Current and future perspectives for improving the outcome of fertility preservation in women

Advancements in the diagnosis and treatment of cancer have increased the number, and improved the prognosis, of cancer survivors. Although embryo or oocyte cryopreservation is the standard method for fertility preservation, OTC has been declared an acceptable alternative [18, 19]. Embryo or oocyte cryopreservation for fertility preservation may not be appropriate in women with cancer due to complicated individual conditions and treatment schedules. OTC-T is a promising option for fertility preservation in both pre-pubertal girls and adult patients with cancer who require immediate treatment. The recovery of endocrine function following re-implantation is well established, and the live birth rate has been steadily increasing. To prevent fertility loss in women with cancer, individualized fertility preservation options must be considered, including the patient’s age, marital status, chemotherapy regimen, and possibility of treatment delay. Effective multidisciplinary oncofertility strategies (Fig. 1), involving the inclusion of a highly skilled and experienced team, freezing-thawing methods, surgical procedures for transplantation, and the latest scientific studies, should be carefully considered for each patient to provide the highest quality of care.
